# β-Amyloid impairs Proteasome structure and function. Proteasome activation mitigates amyloid induced toxicity and cognitive deficits

**DOI:** 10.1101/2024.10.23.619877

**Published:** 2025-02-04

**Authors:** Kanisa Davidson, Mehar Bano, Danitra Parker, Pawel Osmulski, Maria Gaczynska, Andrew M. Pickering

**Affiliations:** 1 Department of Neurology, University of Alabama at Birmingham, Birmingham, AL 35294, USA.; 2 Department of Integrative Biology and Pharmacology, The University of Texas Health Science Center at Houston, Houston, TX 77030, USA; 3 Department of Molecular Medicine, UT Health San Antonio, San Antonio, TX 78229, USA; 4 Institute on Aging, The University of Texas Health Science Center at Houston, Houston, TX 77030, USA

## Abstract

**Background:**

Alzheimer’s Disease (AD) is the leading cause of dementia globally, affecting around 50 million people and marked by cognitive decline and the accumulation of β-amyloid plaques and hyperphosphorylated tau. The limited treatment options and numerous failed clinical trials targeting β-amyloid (Aβ) highlight the need for novel approaches. Lowered proteasome activity is a consistent feature in AD, particularly in the hippocampus. Impaired proteasome function in AD is hypothesized to stem from direct inhibition by β-amyloid or hyperphosphorylated tau, disrupting critical neuronal processes such as memory formation and synaptic plasticity.

**Objectives:**

This study tests the hypothesis that AD related deficits are driven in part by impaired proteasome function as a consequence of inhibition by Aβ. We evaluated how proteasome function is modulated by Aβ and the capacity of two proteasome-activating compounds, TAT1–8,9-TOD and TAT1-DEN to rescue Aβ-induced impairment in vitro, as well as survival deficits in cell culture and Aβ-induced cognitive deficits in Drosophila and mouse models.

**Results:**

Our study demonstrates that oligomeric β-amyloid binds to the 20S proteasome and impairs its activity and conformational stability. The oligomers also destabilize the 26S proteasome to release the free 20S proteasome. Treatment with proteasome activators TAT1–8,9TOD and TAT1-DEN rescue the 20S proteasome function and reduces cell death caused by Aβ42 toxicity in SK-N-SH cells. In Drosophila models overexpressing Aβ42, oral administration of proteasome agonists delayed mortality and restored cognitive function. Chronic treatment with TAT1-DEN protected against deficits in working memory caused by Aβ42 in mice and in hAPP(J20) mice with established deficits, acute TAT1-DEN treatment significantly improved spatial learning, with treated mice performing comparably to controls.

**Conclusions:**

Aβ has dual impacts on 20S and 26S proteasome function and stability. Proteasome activation using TAT1–8,9TOD and TAT1-DEN shows promise in mitigating AD-like deficits by protecting against amyloid toxicity and enhancing proteasome function. These findings suggest that targeting proteasome activity could be a viable therapeutic approach for AD, warranting further investigation into the broader impacts of proteasome modulation on AD pathology.

## INTRODUCTION

Alzheimer’s Disease (AD) represents the leading cause of dementia, with approximately 500,000 new cases a year, and 50 million people worldwide are living with AD. The disease is marked by progressive declines in cognitive and executive function, with accumulation of β-amyloid plaques and hyperphosphorylated tau as key pathologic markers. There are very limited treatment options for AD, making the development of interventions to slow or reverse AD symptoms a critical area of research. This growing need is challenged by the negative experience of numerous failed clinical trials aimed at a single specific target, β-amyloid (Aβ) or pathways surrounding Aβ processing. This driving the need for novel targets and approaches

Work by our team and others shows lowered activity of the proteasome as a robust feature of AD, described in animal models of AD and in post-mortem brains from AD patients (2–6). In patients with AD, proteasome impairment appears most pronounced in the hippocampus and parahippocampal gyrus, with some impairment in the middle temporal gyri, and inferior parietal lobule. It is thought that impaired proteasome function in AD stems from direct inhibition of the proteasome by β-amyloid or hyperphosphorylated tau (4, 5, 7). It is suggested that β-amyloid may act as an allosteric inhibitor, preventing opening of the substrate gate of the 20S core and thus impairing the uptake of substrates by the proteasome assemblies, however this has not been explored in detail (8). In patients with AD, levels of proteasome impairment correlated with levels of β-amyloid (6) and hyperphosphorylated tau (9).

The proteasome system plays critical roles in many functions of the nervous system. These include the formation and strengthening of memory through the promotion of long-term potentiation by augmentation of the CREB transcription factor – either by degrading repressors of CREB or subunits of activators, which repress their function (10–18). The proteasome also plays a key role in dendritic spine formation. The proteasome is sequestered in the dendritic spine through signalling by CaMKIIα and the NMDA receptor and is thought to then cause local degradation of factors that repress dendritic spine growth. The result is increased growth of dendritic spines (19–23). As a consequence, impairment of proteasome function produces deficits in long-term potentiation, dendritic spin density, memory formation and consolidation. Based on this and the data we will describe below we hypothesize that proteasome impairment in AD is a driving factor of AD pathology (24–29).

In prior work we report proteasome augmentation to delay Alzheimer’s disease like deficits via overexpression of a rate limiting proteasome subunit (PSMB5) in cell culture, Drosophila and mouse models of AD (6). Other groups have made similar findings in C. elegans models of AD (30). Furthermore, we developed a set of novel proteasome activating compounds based on non-toxic and non-immunogenic cell-penetrating peptides (CPPs) used in clinic as safe drug vehicles. Our two lead compounds which we have termed TAT1–8,9TOD and TAT1-DEN are built from an N-terminal activity anchor penetrating the pocket between α1 and α2 proteasome subunits and connected via a β-turn to a specificity clamp interacting with the proteasome core particle’s “α face” (6, 31, 32). Our leads augment activity of both the core proteasome (20S) and the 26S proteasome holoenzyme,up to 9-fold and 3-fold, respectively (5, 31). Further we report our leads to protect against AD-like deficits in cell culture and Drosophila with pilot data supporting a protective effect in mice (6). In our previous work we reported that AD protective effects to stem partly from modulation of amyloid precursor proteins (6).

In this study we describe how proteasome function is modulated by Aβ and show AD protective effects from proteasome modulation to occur through protection against amyloid toxicity. We demonstrate β-amyloid to modulate 20S conformation, altering its function and to destabilize 26S proteasome. Treatment of 20S proteasome with the activators at least partially rescues from the conformational and activity defects. We then show that proteasome impairment increases sensitivity to amyloid toxicity and that both chronic and acute proteasome treatment with proteasome activating compounds can rescue AD-like cognitive deficits in Drosophila and mouse AD models.

## RESULTS

### 1. Oligomeric Aβ42 inhibits the 20S proteasome and alters its conformational equilibrium. The effects are mitigated by the proteasome activators. alters proteasome gate conformation.

**Fig. 1.**
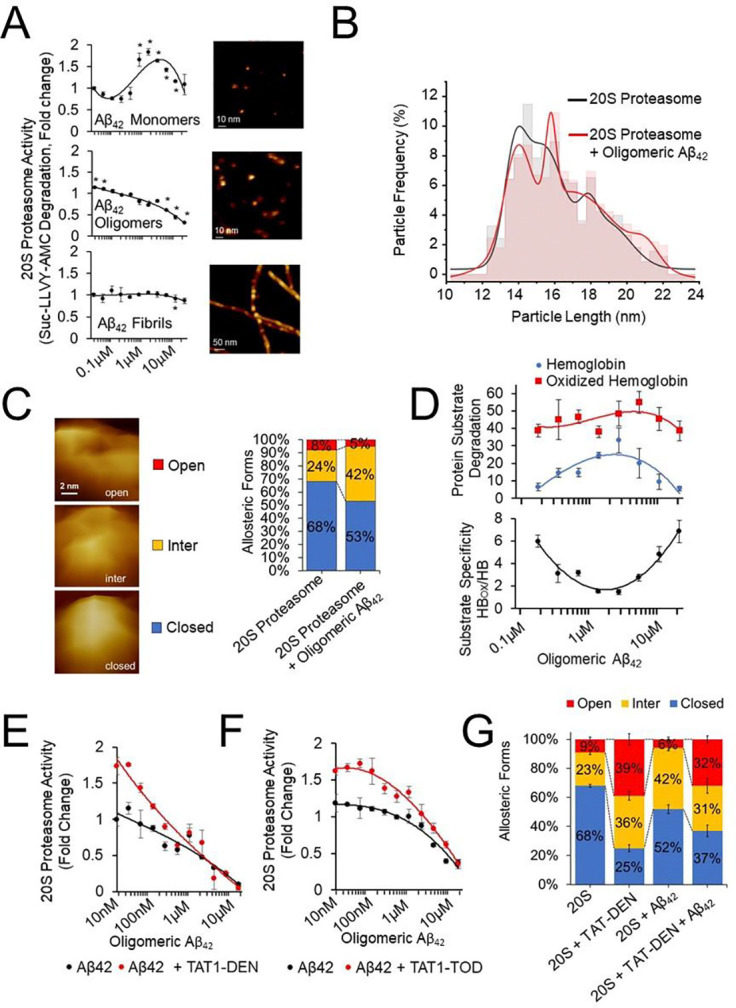
Oligomeric Aβ42 impairs 20S proteasome activity and conformational stability. The impairment can be mitigated by proteasome activators. **(A)** 20S Proteasome chymotrypsin-like peptidase activity is inhibited by oligomeric Aβ42, but not by Aβ42 monomers or fibrils. N = 4. Asterisks denote statistically significant differences (p<0.05). Right: atomic force microscopy (AFM) images of Aβ particles (tapping mode in air). The occasional larger particles in the “monomer” preparation are likely spontaneously forming oligomers. (**B**) Morphometric analysis of the 20S proteasome particles imaged by AFM (tapping mode in liquid) reveals shifts in the particles’ dimensions upon incubation with oligomeric Aβ42. 827 control 20S particles (incubated with a vehicle) and 1181 particles incubated with 2 μM oligomeric Aβ42 were analysed. Solid lines are fittings for the frequencies of control (black) and oligo-treated (red) particles. Since almost all particles are in to-view position, the “length” parameter generated during the particle analysis corresponds to the diameter of the 20S α face. The diameters are raw numbers without correction for tip broadening. When the correction of 2 pixels for SNL probe is applied, the diameter for peak 1 (raw: 14 – 15 nm) falls into 10 – 11 nm range, in excellent agreement with the crystal structure of the human 20S proteasome (1). See Results for putative assignment of proteasome forms to the numbered peaks. **(C)** Incubation with oligomeric Aβ42 shifts the conformational equilibrium of 20S core particles imaged by AFM (tapping mode in liquid) toward less open-gate and closed-gate forms, but more intermediate forms. **(D)** Oligomeric Aβ42 does not significantly affect degradation of oxidized hemoglobin. Degradation of hemoglobin is enhanced by a range of oligomeric Aβ42 concentrations. N=4 samples. (**E, F**) Treatment of the 20S proteasome with activators TAT1-DEN or TAT1-TOD partially protects from inhibition inflicted by the oligomeric Aβ42. (**G**) Incubation with the proteasome activator TAT1-DEN induces a dramatic shift toward open-gate forms, even in the presence of 2 μM of oligomeric Aβ42. The numbers in columns indicate percent of conformers. The number of particles analyzed: 733 (vehicle control), 843 (with oligo Aβ42), 270 (with 1 μM TAT1-DEN) and 171 (with oligo Aβ42and TAT1-DEN). Average ± SD, n= 5 to 9 fields.

Proteasome function is robustly impaired in animal models of AD and in post-mortem brains from AD patients (2–6). This effect is driven by interaction of the proteasome with β-amyloid or hyperphosphorylated tau leading to its inhibition (4, 5, 7). As demonstrated in Fig. 1A, only Aβ42 oligomers consistently inhibited the chymotrypsin-like activity of the purified human 20S proteasome. Surprisingly we also observed a mild but reproducible “activation” of the 20S at very low doses of oligomeric Aβ42 (20nM-40nM) and high doses of monomers (500nM – 10 μM; Fig 1A). Those may stem from complex interactions of in situ-formed oligomers of distinct sizes, including very small oligomers in the monomer preparation, with the proteasomes. We found fibrillar amyloid to show no substantial change in activity (either positive or negative).

To test the mode of amyloid interactions with 20S proteasomes, we examined the effect of oligomeric Aβ42 on length (diameter) distribution of purified human 20S proteasomes using Atomic Force Microscopy (Fig. 1B). The proteasomes were attached to a flat mica surface via electrostatic interactions in the top standing position exposing their α ring to imaging with an AFM probe. Proteasomes length was grouped into four overlapping peaks with maxima at 13.8, 15.3, 17.8 and 18.9 nm (Fig. 1B). We traced the particles assembled in the following three peaks as mostly representing closed, intermediate, and open gate conformations. The fourth peak contained mostly misshaped particles and occasional side-view (lying) proteasomes. The presence of Aβ42 oligomers shifted the position of length maximum for peak 2 to 15.8 nm and peak 4 to 21 nm, and decreased the diameter of particles in peak 3 to 16.9 nm. The presence of oligomers limited the number of the smallest particles (peak 1; closed-gate) and increased enumeration of larger proteasomes (peaks 2–4) that contain substantial number of intermediate proteasomes likely decorated with Aβ42 oligomers, further increasing the effective length of particles, especially for peak 4 (Fig. 1B). Classification of proteasome particles by a single particle analysis into closed-gate, intermediate and open-gate conformers revealed that the presence of oligomeric Aβ42 decreased the content of closed conformation from 68 to 53% but increased content of intermediate proteasomes from 24 to 42% (Fig. 1C). At the same time the content of open gate proteasome remained unchanged (a nonsignificant decrease; Fig. 1C).

Because of the small size of the Suc-LLVY-AMC proteolytic substrate we hypothesized that the intermediate gate conformation produced may have made entry easier for the substrate explaining the apparent increase in activity at low doses of oligomeric Aβ42. Next, we tested degradation of fluorescently (AMC) labelled haemoglobin by the core proteasome (Fig. 1D). The 20S proteasome is expected to selectively degrade oxidized over native proteins (33, 34). No significant inhibition was noted for degradation of oxidized haemoglobin (Fig. 1D). Interestingly, incubation of the 20S proteasome with oligomeric Aβ42 at a range of concentrations from about 500nM to 10μM resulted in a noticeable increase in degradation of haemoglobin, the protein that, without at least a partial unfolding, is not cleaved by the 20S proteasome at a significant rate (34). Since the accessible carboxyl groups of hemoglobin were blocked with the AMC, its structural integrity may have been compromised, albeit not as prominently as in the case of oxidized hemoglobin, used as a model stress related protein substrate (34). We may also speculate that complex interactions of Aβ42 oligomers with the heme and the globin lead to a partial unfolding of the protein. Regardless of the origin of apparent accessibility of the globin to the proteasome, the transient “activation” for AMC-hemoglobin degradation without a significant change in oxidized AMC-hemoglobin degradation may suggest oligomers-related change in the proteasome selectivity for substrates, before the increasing concentration of oligomers would effectively suppress the degradation (Fig. 1D).

### 2. Aβ_42_ Proteasome inhibition and gate conformation is mitigated by the proteasome activators.

Next, we tested how the proteasome activation may impact the putative interactions of the 20S with oligomers. We developed a set of proteasome peptidomimetic activators TAT1–8,9TOD (TAT1-TOD) and TAT1-Dendrite (TAT1-DEN) which show a robust ability to enhance both 20S and 26S proteasome function *in vivo* and *in vitro* a detailed characterization is reported in our prior publications (6, 31, 32). Importantly we show that our agonists are able to still enhance proteasome function in the presence of oligomeric Aβ42. Both TAT1-TOD and TAT1-DEN demonstrated diminishing activating capacity at higher oligo Aβ42 concentrations suggesting competition with amyloid (Fig 1E, F). Importantly, utilizing AFM we found that TAT1-DEN shifts the equilibrium of conformers to limit enumeration of closed molecules to only 25% and increase content of open molecules to 39%. Surprisingly, proteasomes treated with oligomeric Aβ42 and TAT1-DEN showed the distribution of the confromers similar to those treated with Tat-DEN alone. This observation suggests that TAT1-DEN prevents Aβ42 induced formation of the excess of intermediate conformers and supports formation of open proteasomes essential for accepting substrates.

### 3. Oligomeric A 42 drives 26S proteasome disassembly

**Fig. 2.**
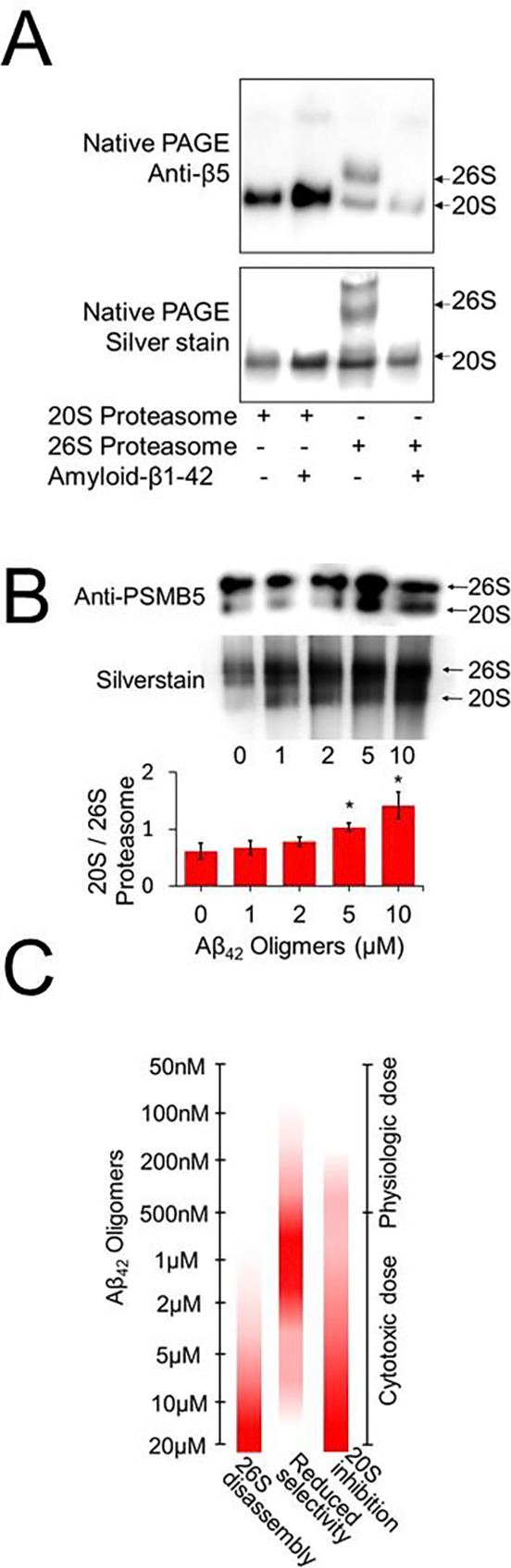
Oligomeric Aβ42 destabilizes 26S proteasome. (A) Native page immunoblot depicting purified 20S and 26S proteasome under incubation with oligomeric Aβ42. Immunoblot performed against proteasome β5 subunit and accompanying total protein silver stain. Arrows depict 26S and 20S proteasome assemblages. (B) Native page immunoblot depicting purified 26S proteasome under incubation with varying concentrations of oligomeric Aβ42. Top image shows a representative set, histogram represents N=3 per condition. (C) Model for impact of Aβ on proteasome processes. *p < 0.05, Student’s t test was used unless otherwise stated. N represents the number of animals or samples per group.

We next examined function of the 26S proteasome which is composed of the 20S core with a 19S regulatory cap facilitated degradation of polyubiquitinated proteins (35). We found Aβ oligomers to produce a destabilization of the 19S cap. Through native PAGE immunoblotting we showed Aβ oligomers when incubated with purified 26S proteasome to produce a decline in proteasomes with a bound 19S cap and an increase in free 20S proteasomes (Fig 2A). We observe a progressive effect with increasing concentrations of Aβ42 producing increased 26S proteasome disassembly (Fig. 2B). Combined we hypothesize that amyloid impairs 3 critical aspects of proteasome function: 20S Activity, 20S substrate specificity and 26S destabilization (Fig. 2C).

### 4. Proteasome inhibition increases in cellulo Aβ toxicity and proteasome activators protected against in Aβ-induced cell death.

**Fig. 3.**
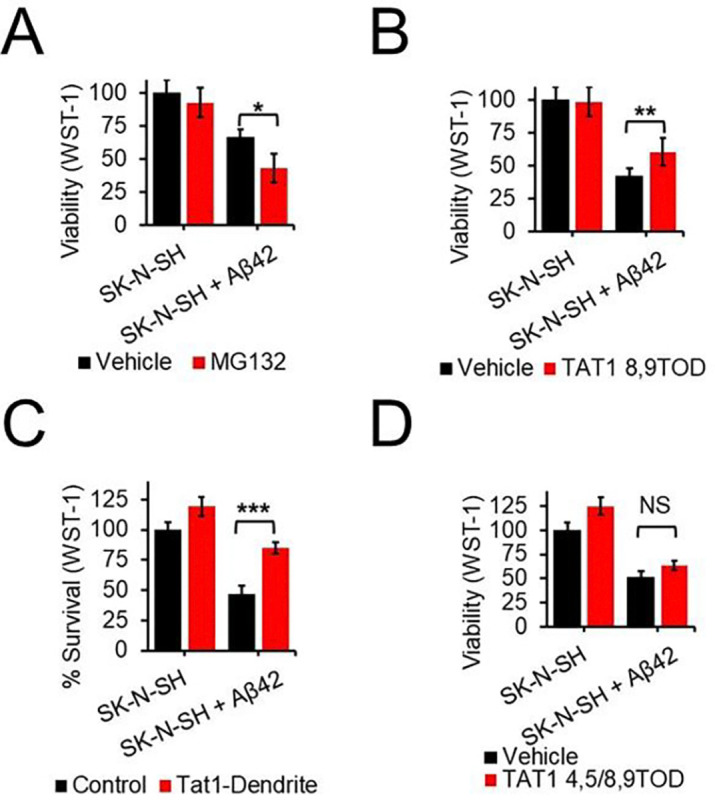
Proteasome inhibition and activation modulate in cellulo Aβ toxicity. WST1, cell viability assays in SK-N-SH cells seeded at 100,000 cells per ml after 4 days treatment with 5μM oligomeric Aβ42. Cell death was modulated under co-treatment with (A) 0.1μM Mg132, (**B**) 1μM TAT1–8,9TOD, (**C**) 1μM TAT1-DEN, (**D**) 1μM TAT1–4,5/8,9TOD. N = 15 wells/condition. *p < 0.05, **p < 0.01, and ***p < 0.001. Student’s t test was used unless otherwise stated. N represents the number of animals or samples per group. NS, not significant.

Using neuroblastoma SK-N-SH cells we demonstrate that proteasome inhibition increases susceptibility of cells to Aβ toxicity. Treatment with the proteasome selective inhibitor Mg132 does not produce cell death in itself at our experimental dose but cotreatment with oligomeric Aβ42 and Mg132 produces greater cell death than treatment with oligomeric Aβ42 alone (Fig. 3A). We found that augmentation of proteasome function through treatment with our agonists protects against Aβ42 toxicity. Treatment of cells with our proteasome agonists TAT1–8,9TOD or TAT1-DEN both reduce cell death as a consequence of Aβ42 (Fig. 3B–C). Furthermore, we show the impacts of our proteasome activators on Aβ toxicity are driven directly as a consequence of proteasome activation. Using TAT1 4,5/8,9TOD a structurally similar peptidomimetic but with poor function as a proteasome activator we show no significant protection against Aβ42 toxicity (Fig. 3D). These results suggest the impacts on Aβ toxicity to stem directly from the role of our compounds as proteasome activators.

### 5. Proteasome activators protect against Aβ related cognitive and survival deficits in Drosophila

**Fig. 4.**
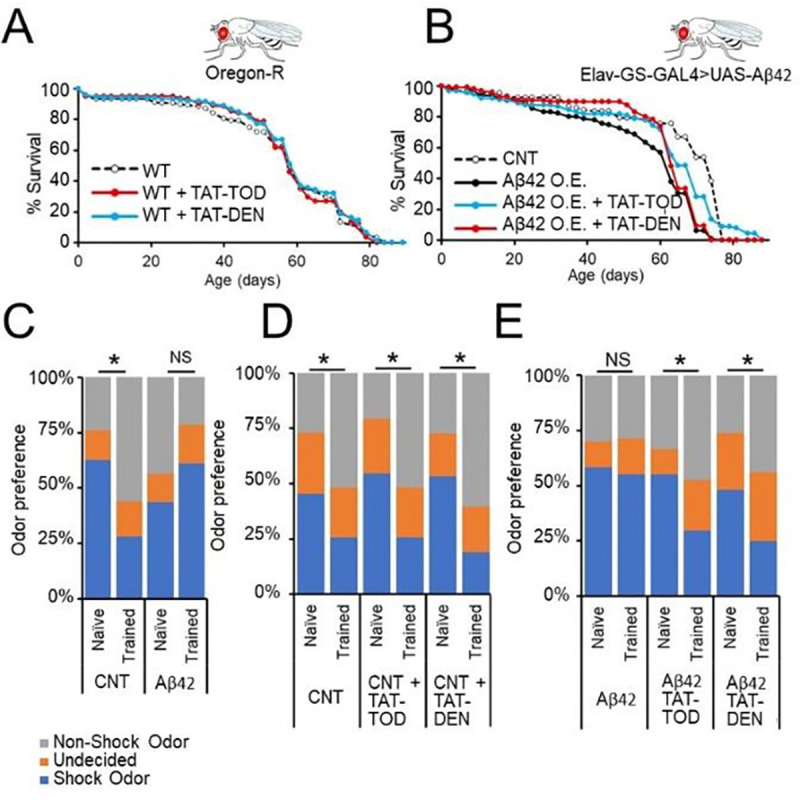
Proteasome augmentation rescues survival and cognitive deficits in a Drosophila Aβ42 overexpression model. (A,B) Survival assay in mated female Oregon-R flies (A) or Elav-GS-GAL4>UAS-Aβ42 flies (B) in the presence of 1μM TAT1–8,9TOD / TAT1-DEN or vehicle mixed directly into food. N = 89–150 animals/condition. (C-E) Olfaction aversion training assay in day 30 comparing Elav-GS-GAL4>UAS-Aβ42 ± 200 RU486 (CNT/Aβ42 O.E.) in the absence and presence of 1μM TAT1–8,9TOD / TAT1-DEN or vehicle mixed directly into food. *p < 0.05, **p < 0.01, and ***p < 0.001. Significance is based on Log-rank test in A,B and Chi-Sq test in C-E. N represents the number of animals or samples per group. NS, not significant.

We next sought to determine if proteasome augmentation would protect against toxicity effects of Aβ in an animal model. We have previously report that augmentation of proteasome function modulates the formation of Aβ through impacts on amyloid precursor machinery (6). To determine if there are downstream protective impacts independent of Aβ synthesis we utilized a Drosophila model of Alzheimer’s disease which overexpresses pre-formed Aβ42 under control of a pan-neuronal Elav driver (Elav-GS-GAL4>UAS-Aβ42). This model experiences survival and cognitive deficits. We first demonstrated treatment with our proteasome agonists TAT-TOD and TAT-DEN to have no significant impact on lifespan in wild-type (Orgeon-R) flies (Fig 4A). While we found oral administration of either TAT-TOD or TAT-DEN to delay mortality as a consequence of Aβ42 overexpression (Fig. 4B). We found TAT-DEN to delay only early life toxicity effects from Aβ42 while TAT1–8,9TOD to delay both early and late life toxicity effects (Fig. 4B). To investigate the impacts on cognition, a key aspect of AD pathology, we employed an olfaction aversion training assay. Flies are exposed to two distinct odors while given an electric shock under exposure to one. The flies were then placed in a 2-sided chamber with the two odors released from either side and a set of doors in the middle. Aversion to the odor associated with an electric shock was quantified as a measure of learned response. We demonstrate control flies to show a training induced aversion to the odor associated with an electric shock, but this is lost in flies which overexpress Aβ42 (Fig. 4C). Treatment of control flies with proteasome agonists produced no effect on training induced odor avoidance (Fig. 4D). However, treatment of flies which overexpress Aβ42 with either TAT-TOD or TAT-DEN restored training induced odor avoidance (Fig. 4E).

### 6. Acute and chronic treatment with Proteasome activators protects against Aβ related cognitive and deficits in mice

**Fig. 5.**
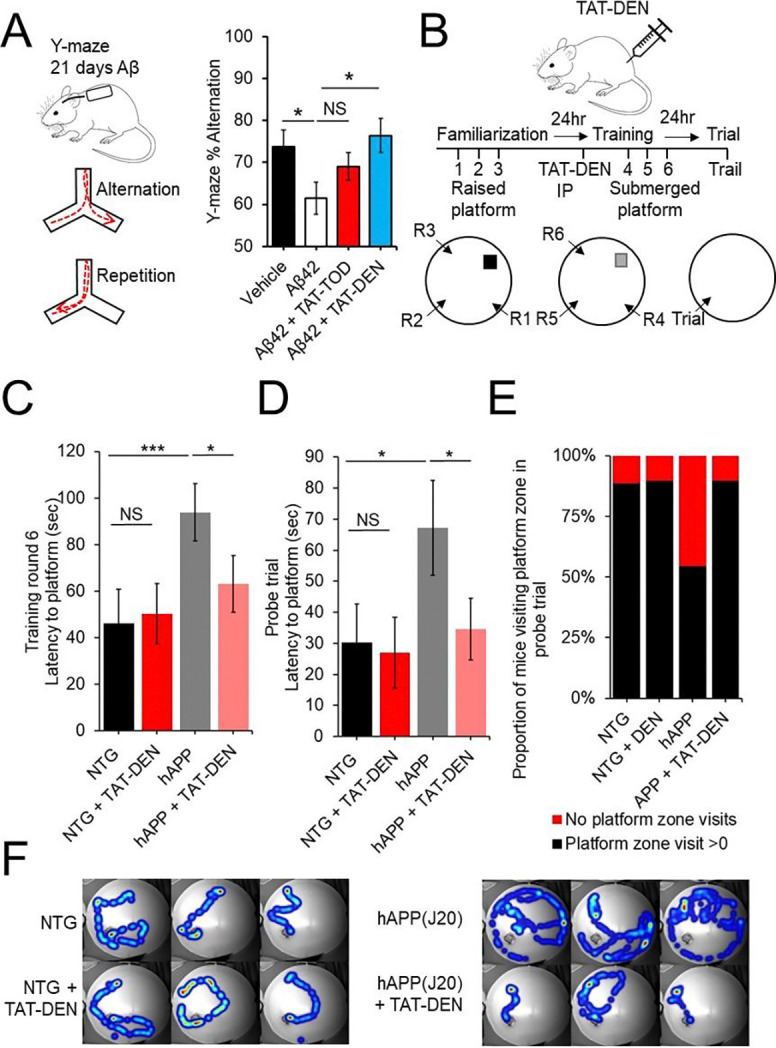
Aβ produces deficits in working memory, memory formation and acquisition which may be rescued by either chronic or acute treatment with proteasome agonists. (A) Osmotic pumps surgically implanted in 3 months old C57BL6J mice to deliver to the hippocampus +/− 1.25μmol of Aβ42 +/−100pmol of TAT-TOD/DEN per hr for 21 days. Spontaneous alternation in Y-Maze assessed. N=11 animals/group. (B) Morris water maze in 18 month old hAPP(J20) and littermate controls. Animals received 3, 1 minute training sessions from different locations on day 1 with a raised platform. Animals were IP injected on Day 2 with 400nmol/kg of TAT-DEN or an equivalent volume of vehicle to produce an approximate blood concentration of 5μM. Animals we allowed 30 minutes recovery then subjected to 3 training rounds with a submerged platform. A probe trail was performed 24hr later N = 9–11 per group. (C) Shows latency to the platform on the final training session. (D) Shows latency to platform location in the probe trial. (E) shows the proportion of mice entering platform zone during probe trail. (F) representative heatmaps (3 per group) of final training session, recording was stopped once animals reached platform. Student’s t test was used unless otherwise stated. N represents the number of animals or samples per group. NS, not significant.

We next investigated mammalian translational relevance, whether proteasome augmentation can protect against Aβ induced cognitive deficits in mice. To do this we employed mice fitted with an osmotic pump to deliver either Aβ42 and vehicle or Aβ42 in conjunction with TAT-TOD or TAT-DEN directly to the brain for 21 days. Treatment with Aβ42 produced deficits in working memory in mice as assessed by arm alternation in a Y-maze assay (Fig 5A). We showed co-treatment with TAT-DEN to significantly improve alternation back to wild type levels. Treatment with TAT-TOD also produced improvements but unlike TAT-TOD these did not reach statistical significance (Fig 5A). For this reason we focused our subsequent investigations on TAT-DEN.

Previous studies have shown acute treatment with proteasome inhibitors to produce deficits in long term memory formation and retrieval (24, 26, 27). Our running hypothesis is that Aβ induced inhibition of proteasome function may contribute to deficits in memory formation. We thus hypothesized that acute treatment with proteasome activators might rescue AD-like deficits in memory formation. To test this hypothesis, we employed 18-month-old hAPP(J20) along with littermate controls. Animals of this age were utilized so as to ensure pronounced AD-like deficit at the start of the experiment.

Animals were evaluated for spatial learning and memory via a modified version the of Morris water maze. We employed a design similar to the design employed by Artinian and colleagues (26) where they performed familiarization of animals to the maze on day 1, acquisition training on day 2 followed by injection of a proteasome inhibitor and a probe trial on day 3. In this design they reported treatment with the proteasome inhibitor to produce spatial memory deficits (26). In our design, animals were first acclimated to the maze using a raised platform at multiple entry locations on day 1. On day 2 animals received an IP injection of TAT-DEN then 3 training sessions at multiple entry sites with the platform submerged. On day 3 a probe trial was performed (Fig. 5B). We demonstrated that hAPP(J20) mice show deficits in memory formation showing reduced latency to find the submerged platform in the final training session, while hAPP(J20) mice treated with TAT-DEN showed improvement in this measure. TAT1-DEN produced no effect on memory acquisition in control mice (Fig 5C). Furthermore, we show TAT1-DEN to rescue deficits in memory retention. hAPP(J20) mice showed deficits during the next day probe trial with deficits in latency to reach the platform zone and fewer hAPP(J20) mice entered the platform zone, in comparison to control animals. In contrast hAPP(J20) mice treated with TAT-DEN showed similar latency to the platform zone and likelihood to enter the platform zone as control animals (Fig 5D–E). Fig 5F shows example heatmaps for the final training session. Recordings were stopped once the mouse arrived at the platform zone.

Our results demonstrate Aβ impacts on learning memory and cell death to be in part dependent on its role as a proteasome inhibitor and that proteasome activation can rescue AD-like cognitive deficits both over chronic and acute treatment.

## DISCUSSION

Our study makes a number of key findings. Prior research has reported Aβ to act as a proteasome inhibitor (2–6). We confirm the notions and propose a molecular mechanism behind the complex phenomena of activity impairment. We find that indeed the oligomeric Aβ42 is an inhibitor while fibrillar amyloid appears ineffective at proteasome modulation. Importantly, we find the oligomeric Aβ binds to the 20S core and induces shifts in the partition of proteasome gate conformers, with excessive representation of the intermediate form, which is neither fully closed nor fully open but apparently does not support the increased activity. In addition, we find that Aβ42 induces a disassembly of the 26S proteasome in favor of the 20S proteasome. The 20S proteasome selectively degrades oxidized protein and is important for oxidative stress response and the clearance of damaged proteins (33, 34). However, we demonstrate that the 20S core in the presence of Aβ oligomers may display non-standard selectivity toward non-oxidized disordered protein substrates, possibly further compromising proteostasis before the soluble oligomers would ultimately compromise the 20S proteasome’s actions. The prevailing narrative for how proteasome dysfunction will modulate cognitive deficits is through programmed control of such pathways as the CREB transcription factors modulating long term potentiation (10–18), and modulation of the NMDA receptor impacting growth of dendritic spines (19–23). These functions would occur through turnover of polyubiquitinated proteins by the 26S proteasome, not the 20S proteasome. A potential explanation for this finding comes from work showing a novel plasma membrane proteasome in neurons. The authors report that approximately 40% of proteasome (specifically 20S proteasome) in neurons have a plasma membrane localization, which was not observed in non-neuronal cell cultures (36, 37). The authors go on to show that impairment of these plasma membrane proteasome impairs calcium signalling, thus affecting key neuronal processes in neuronal function (37, 38). As such it is possible that AD-like deficits produced by proteasome inhibition form Aβ and its subsequent correction through proteasome augmentation are from impacts to the plasma membrane proteasome.

Our data shows proteasome activating compounds TAT1–8,9TOD and TAT1-DEN as potent protectors against AD-like deficits. The findings complement and expand on our prior work. We have reported previously that our agonists enhances proteasome activity in human neuroblastoma SK-N-SH cells, prevent cell death in a TET-OFF APP^N17,C99^ overexpression (MC65) cell line. We demonstrated that flies which overexpressed amyloid precursor proteins, which experience cognitive and survival deficits, had reduced deficits under treatment with our agonists. We also reported pilot data of improvements in spatial learning and memory in hAPP(J20) mice treated with proteasome agonists over a 2 week period, we also demonstrated our compounds to have acceptable to excellent serum stability and BBB penetrance (6). We have now expanded on this work showing protection in an additional cell culture model, an additional Drosophila model and a much more robust demonstration of protection in multiple mouse models. This positions our leads as promising targets for clinical translation.

In our prior studies we suggested that the protective impacts from proteasome augmentation stem from increased turnover of amyloid precursor machinery thus slowing AD progression (6). In this study we employed models which involved either established deficits or treatment with preformed amyloid. This was undertaken intentionally to separate impacts of amyloid synthesis from amyloid toxicity. Indeed, we demonstrated that protective effects still exist both under acute treatment in animals with preformed deficits and in models treated with pre-formed amyloid. This β-amyloid produces inhibition of proteasome function which is important in a myriad of critical neuronal processes such as long term potentiation, dendritic spine stability, axon growth, synaptic vesicle transport etc (39). We hypothesize that impairment of the proteasome’s role in these processes contributes to AD pathology which is corrected under treatment with proteasome agonists. Critically our data shows acute treatment to produce rapid rescue of AD-like deficits suggesting proteasome augmentation may viable as a therapeutic intervention. Notably of our two leads we found more robust effects from TAT-DEN than TAT-TOD. We have previously reported TAT-DEN to possess greater serum stability as well as a larger effective dose range which may explain these differences.

Notably this investigation was focused on amyloid, however tau is also highly impactful to AD and to proteasome function. Levels of proteasome impairment correlated hyperphosphorylated tau (9) similar to Aβ. Further investigation is needed to uncover the impacts of proteasome modulation on tauopathy and tau toxicity.

In conclusion, we demonstrate proteasome activation to rescue AD life deficits and to protect against detrimental impacts from Aβ. Our proteasome-activating compounds, TAT1–8,9TOD and TAT1-Dendrite, show promise in mitigating AD-like deficits across multiple models, supporting their therapeutic potential. These results underscore the importance of targeting proteasome function to address both amyloid and tau-related AD pathology, warranting further investigation into proteasome modulation’s broader impacts.

## METHODS

### Key reagents.

Purified 20S Proteasome (R&D Systems, Cat# E-360), purified 26S Proteasome (R&D Systems, Cat# E-365). TAT1-TOD and TAT1-DEN were generated as described in out prior study (31, 32)

### Preparation of Aβ.

Purified Aβ42 (Genscript, Cat# RP20527–1), was solubilized in 1% NH_3_ at 12.5mg/ml then diluted to 1mg/ml in PBS. For monomers, samples were sonicated for 60 minutes, vigorously vortexed for 1 minute then centrifuged at 16,000g for 10 minutes to remove aggregates. For oligomers, samples were sonicated for 60 minutes, incubated at 4°C for 24hr under gently agitation, vigorously vortexed for 1 minute then centrifuged at 14,000g for 10 minutes to remove aggregates. For fibrils, samples were sonicated for 60 minutes, vigorously vortexed for 1 minute then incubated at 37°C for 24hr. Consistent with prior studies (40). The size distribution of the oligomers in the supernatant was examined by atomic force microscopy (AFM).

### Fly lines and strain maintenance

UAS-Aβ42 (Cat# 64216), was obtained from the Bloomington Drosophila Stock Center (NIH P40OD018537). Elav-GS-GAL4 and Orgeon-R stocks were kindly donated. All lines were maintained on agar-cornmeal-dextrose-yeast growth medium (41) in a humidified 25°C incubator with 12-hour light/dark cycles. All crosses were set up with female virgins of the respective GAL4 driver line and male UAS-Aβ42 or W^1118^ flies. Progeny were collected within 48 hours of eclosion and allowed to mate on 10% sugar/yeast (SY10) medium (41) for another 48 hours. Females were then separated and sorted into sets of 25 flies per vial containing SY10 medium supplemented with either 200 μM mifepristone (RU-486) or ethanol vehicle, mixed directly into the food. Blue dye #1 (8 μM) was added to food containing RU-486 for the purpose of identification. Carbon dioxide was used to briefly anesthetize flies for sorting. Flies were moved to vials of fresh medium every 2 to 3 days.

### Mice

hAPP(J20) mice were maintained by heterozygous crosses with C57BL/6J mice (the Jackson Laboratories, Bar Harbor, ME). Non-transgenic littermates were used as controls. Mice were housed in ventilated cage racks with up to seven animals per cage under 12-hour light/dark cycles at 24°C. Mice were monitored daily by University of Alabama at Birmingham Laboratory Animal Resources staff and were transferred to new cages weekly.

### Cell culture

SK-N-SH Cells were cultured in EMEM (Eagle’s Minimum Essential Media) supplemented with 10% heat-inactivated fetal bovine serum and antimicrobials [penicillin (100 U/ml), streptomycin (100 μg/ml), and amphotericin B (0.25 μg/ml); Gibco-Invitrogen). Incubators were maintained at 5% CO2 and 37°C. Medium was replaced every 3 to 4 days. For most experiments, cells were seeded at 100,000 cells/ml in either 6-well or 96-well plates 24 hours before assay. In most cases, medium was replaced with serum-free Opti-MEM medium 24 hours before assay.

### Proteasome activity assay

The chymotrypsin-like activity was tested with 50 μM Suc-LLVY-AMC model substrate, in a 96-well plate format, as previously described (6). The reaction buffer consisted of 50 mM Tris-HCl (pH 7.8) + 100mM KCl. For testing the activity of the 26S holoenzyme, the 50 mM Tris-HCl buffer was supplemented with “MAD”: 1 mM MgCl2, 2 mM ATP, and 1 mM DTT (dithiothreitol).

### AMC labelling of protein substrates

Detailed procedures outlined in (42). Hemoglobin, was dissolved in 0.1 M Hepes buffer at 5mg/ml. Followed by addition of 500 μM AMC and 20 mM sodium cyanoborohydride. Solutions were incubated at room temperature for 2 hr. To induce oxidation some samples were incubated with 1mM Hydrogen Peroxide for 1hr. Samples were extensively dialyzed though a 10,000 MWCO centrifugal filter, and a buffer exchange was performed with proteolysis buffer (50 mM Tris–HCl, pH 7.8, 100mM KCl).

### Atomic Force Microscopy (AFM) Imaging

Monomers, oligomers and fibrils of Aβ were imaged using the tapping (oscillating) mode in air with TESP probes and a scanner E of the Multimode Nanoscope IIIa (Bruker Inc., Santa Barbara, CA), as previously described for tau (43). Live 20S proteasome particles were imaged with the tapping mode in liquid using our established procedure (31, 35), with SNL (Sharp Nitride Lever; Bruker) probes. Preparations of proteasomes were mixed with Aβ oligomers (2 μM) and/or TAT activators (1 μM), or the respective vehicles (0.008% ammonia water for oligomers, 1% DMSO for activators). The mixes were incubated for 1 hour at room temperature before diluting in “imaging buffer” (5 mM Tris-HCl, pH 7) and depositing on muscovite mica for electrostatic attachment followed by scanning and imaging in height mode, as described (31). The images of 1 μm^2^ fields (512 × 512 pixels) typically contained from 20 to 200 top-view (“standing”) 20S proteasome particles, with the top of a ring (“α face”) covered by a six-pixel scan line fragment and amenable for analysis. After the standard 1^st^ order flattening (Nanoscope Analysis 1.70 software) or line-wise levelling (SPIP 6.013 software; Image Metrology, Denmark) the raw numerical values of pixels of scans across α faces collected with a practical vertical resolution reaching 1 Å were used to discern between the three conformational forms: closed, intermediate and open, depending on the presence of a “dip” (local minimum; open form), a concave function (intermediate form) or a convex function (closed form) (31, 44). Selected fields were scanned multiple times to follow conformational changes in the same set of particles. For the morphometric analysis of 20S particles the Particle Analysis function in SPIP software was used (45). Frequency analysis, peak fitting and statistical analysis was performed with the OriginPro 2019 ((OriginLabs, Northampton, MA).

### Olfactory aversion training

Experiments were performed as described in (46). Animals were exposed (via an air pump) in alternation to two neutral odors (3-octanol and 4-methylcyclohexanol, prepared as a 1:10 dilution in mineral oil) for 5 min under low red light, and a 100-V 60-Hz shock was applied during exposure to one of the two odors. The odor associated with the electric shock was alternated between vials. After three training rounds per odor, animals were given 1 hour to recover then placed in a T maze (CelExplorer Labs) with opposing odors from either side. Flies were allowed 2 min to explore the maze, after which the maze sections were sealed, and the number of flies in each chamber was scored.

### Cell viability

Cells were maintained in clear 96-well plates. On the day of assay, 10 μl of WST-1 reagent (11644807001, Sigma-Aldrich) was added to each well, and cells were incubated in a 37°C, 5% CO2 incubator for 2 to 4 hours. Absorbance was measured at 450 nm using a Gemini series spectrophotometer.

### Osmotic pump implantation

Adapted from (47), animals were anesthetized with vaporized halothane, and a micro-osmotic pump (Alzet #2004) was stereotaxically implanted into the right lateral cerebral ventricle (at coordinates−1.0 mmmediolateral and−0.5 mm anterioposterior from Bregma;−1.5 mm dorsal-ventral from skull). Pumps contained either Aβ1–42, Aβ1–42 + TAT-TOD. Aβ1–42 + TAT-DEN or vehicle alone. Pumps were placed under the skin and wounds sutured closed. The pump has an expected releases rate of 0.25 μl/hr for 4 weeks. Pumps were loaded with 55μg Aβ42 for an expected release of 68ng/h and 9.9mg/100ul of TAT-TOD/DEN to produce an anticipated tissue concentration of 100nM.

### Morris water maze

This test provides measures of hippocampal-dependent spatial learning and memory. A 121-cm water maze was used. Animals were given a series of three trials, ~30 min apart, per day to find a submerged platform (~1 cm below water level) in a large tank filled with water made opaque through the addition of white tempera-based nontoxic paint at 23.0° ± 1.0°C, in a room sperated from the operator by a curtain. The pool was surrounded by large panels with geometric black and white designs that serve as distal cues. Maximum trial time was limited to 60 s, whereupon mice were guided to the platform. Mice were allowed to remain on the platform for 5 s and then were gently towel-dried and moved to their home cage under a heating lamp until dry. At the end of training, a probe trial was conducted where the platform was unavailable to measure the retention of the former platform location. The time each animal spent in the quadrant formerly containing the platform and the number of passes over that location provided a measure of memory. Data were collected using TopScan (CleverSys) by operators blinded to genotype.

### Y maze

Working memory is assessed by placing animals in a Y-shaped maze made of white Plexiglas with three arms, with equal angles between all arms. Each animal is placed in an arm of the maze and allowed to move freely around the apparatus, while the sequence and number of arm entries for each animal during a 5-min period are recorded manually by an experimenter blinded to the genotype. The number of spontaneous alternations, which occur when a mouse enters a different arm of the maze in each of three consecutive arm entries (e.g., ABC, CAB, or BCA but not BAB), was counted, and percent alternation was calculated as: (# of spontaneous alternations)/(total arm entries − 2) × 100.

### Study approval

All mouse studies performed were approved by the Institutional Animal Care and Use Committee at the University of Alabama at Birmingham (protocol 22179; Pickering, PI).
